# Mapping Photogenerated Electron–Hole Behavior of Graphene Oxide: Insight into a New Mechanism of Photosensitive Pollutant Degradation

**DOI:** 10.3390/molecules29163765

**Published:** 2024-08-08

**Authors:** Kaijie Ni, Yanlong Chen, Ruiqi Xu, Yuming Zhao, Ming Guo

**Affiliations:** 1 College of Chemistry and Materials Engineering, Zhejiang Agriculture and Forestry University, Hangzhou 311300, China; 2 Department of Chemistry, Memorial University of Newfoundland, St. John’s, NL A1B 3X7, Canada

**Keywords:** graphene oxide, photogenerated electron and hole, photosensitive pollutants, tetracycline removal, degradation pathway

## Abstract

The use of graphene oxide (GO) photogenerated electron–hole (e–h^+^) pairs to degrade pollutants is a novel green method for wastewater treatment. However, the interaction between photosensitive pollutants and a GO–light system remains unclear. In this work, the mechanism of degradation of photosensitive pollutant tetracycline (TC) promoted by GO photogenerated e–h^+^ pairs was studied. Our studies encompassed the determination of TC removal kinetics, analysis of active substances for TC degradation, identification of degradation products, and computational modeling. Clear evidence shows that a new reaction mechanism of enhanced adsorption and induced generation of reactive oxygen species (ROS) was involved. This mechanism was conducive to significantly enhanced TC removal. Kinetic studies showed a first-order behavior that can be well described by the Langmuir–Hinshelwood model. Radical scavenging experiments confirmed that ^1^O_2_, •O_2_^−^, and holes (h^+^) were the main active substances for TC degradation. Electron spin resonance analysis indicated that photoexcited TC molecules may transfer electrons to the conduction band of GO to induce the generation of additional ROS. A major transformation product (*m*/*z* 459) during TC degradation was identified with liquid chromatography–mass spectrometry. Density functional theory calculation indicated a stronger adsorption between TC and GO under photoirradiation. This mechanism of photo-enhanced adsorption and synergistic induced generation of ROS provides a new strategy for the removal of emerging pollutants in water. Overall, the new mechanism revealed in this work expands the knowledge of applying GO to wastewater treatment and is of great reference value for research in this field.

## 1. Introduction

Graphene has received unprecedented attention due to its unique properties and highly promising application [1,2,3]. Graphene oxide (GO), a common precursor for graphene preparation, has additional oxygen-containing functional groups in its structure. These oxygenated groups are covalently bound to the base surfaces (epoxies and hydroxyl groups) and/or edges (carbonyl and carboxyl groups) of GO [4,5], which in turn disrupt the perfect lattice structure of graphene and change the hybrid state of some carbon atoms from sp^2^ to sp^3^. As such, GO attains a relatively large energy gap to exhibit semiconducting properties [6]. Compared with pristine graphene, the covalent oxygenated functional groups present in GO lead to significant structure defects. This is concomitant with some loss in electrical conductivity [7], which limits the direct application of GO in electrically active materials. However, at the same time, the polar oxygen functional groups of GO render it strongly hydrophilic. This gives GO good dispersibility in many solvents, particularly in water, which is important for processing and further derivatization [8]. Usually, GO has larger surface areas than pristine graphite, because the oxygen groups of GO expand the interplanar space. The specific surface area of drying GO reported in the literature ranges from 30 to 295 m^2^·g^−1^ depending on the oxygen contents [9]. Because of its unique properties, GO has been actively researched in modern optoelectronics, ranging from supercapacitors [10] to lithium-ion batteries [11], flexible electronic devices [12], and biomedicines [13]. Moreover, the extended π-conjugated units in the GO structure result in photosensitivity; upon photoexcitation, a π-electron in the sp^2^ domain is promoted to the π* orbital, populating the conduction band with an electron and creating a hole in the valence band. The photochemistry taking place at the sp^2^ domains of GO can be specifically tailored to achieve photocatalytic performance [14]. In addition, the abundant oxygen-containing functional groups of GO make it show strong hydrophilicity. Consequently, GO serves as an ideal nanomaterial to induce photochemical reactivity in aqueous environments.

To date, most of the studies published on the performance of GO in removing contaminants focus on the aspect of photocatalytic degradation of organic pollutants, in which GO was used together with metal [15,16,17,18,19,20,21,22] or non-metal [23,24,25,26,27] catalysts. In these studies, GO was used as a photocatalyst carrier to promote the dispersion of nanoparticles and to enhance the separation efficiency of photogenerated e–h^+^ pairs of the photocatalyst for improved photocatalytic efficiency [28]. Conversely, undoped single GO materials are rarely addressed in the field of photocatalytic degradation of organic pollutants [29,30,31,32]. Nevertheless, some studies have already pointed to this possibility. For example, GO was found to change into reduced GO under light conditions, producing photodegraded products such as carbon dioxide and polycyclic aromatic hydrocarbons [33,34]. Pedrosa et al. reported that GO prepared by the Brodie method shows a high degree of e–h^+^ separation efficiency and can degrade 95% phenol under light irradiation [35]. Recently, Zou et al. demonstrated GO under simulated sunlight irradiation can promote the rapid degradation (95%) of paracetamol (APAP) within 10 min. Their studies revealed that the main active substance for the degradation of APAP comes from the photogenic holes (h^+^) of GO [36]. These studies demonstrate that the photoreactivity of GO can significantly impact the fate and transformation of numerous environmental pollutants. However, the use of the photoreactivity of GO for the degradation of emerging pollutants has not yet been fully explored. More fundamental studies are warranted to acquire a deep understanding of the interplay between GO’s adsorptivity and photoactivity before the potential of GO can be fully unlocked in environmental applications.

Antibiotics represent important emerging contaminants of global concern [37]. TC is one of the most commonly used photosensitive antibiotics in modern medicine [38]. Nowadays, TC is frequently detected in wastewater. The continuous release of TC into the environment is surely an issue of serious environmental and health consequences [39]. Removal of TC from aquatic environments is an urgent task to carry out, but traditional municipal wastewater treatment technologies are ineffective in addressing this issue. TC and other photosensitive antibiotics are chemically stable and not susceptible to biodegradation. The use of photoactive GO to degrade pollutants is an important green technique for wastewater treatment. Since this method only involves GO and photons, it can effectively avert secondary pollution caused by using toxic metal-based photocatalysts. In addition, the electronic and bandgap properties of GO can be regulated through further oxidation or reduction, which are useful for the design of novel highly efficient photocatalysts. To this end, we have recently conducted a series of investigations of the interactions of GO with TC under various conditions. We proposed that GO can act as a dual-functional nanomaterial to promote the efficient removal of antibiotic pollutants from water. On one hand, GO has been known to be an effective adsorbent of antibiotics such as TC [40]. On the other hand, GO may possess the ability to photocatalytically degrade TC, which is evidenced by previous literature reports on the combined use of GO with other photocatalysts to promote the photodegradation of TC [41]. This manuscript, hence, adds new knowledge about the concerted effects of the adsorptive and photoactive sites in GO on the removal of photosensitive pollutants. The prepared GO was fully characterized. The removal of TC by GO under various conditions was performed. Radical scavenging experiments and ESR analysis were conducted. The degradation pathway of TC was proposed based on the LC/MS results. Finally, DFT calculation was used to reveal the adsorption of TC on GO under photoirradiation conditions. A new mechanism of pollutant removal promoted by GO photogenerated e–h^+^ pairs was disclosed.

## 2. Results and Discussion

### 2.1. Characterization

The prepared GO was characterized by a range of spectroscopic and microscopic analyses (Figure S1). The FT-IR spectrum shows the presence of O–H (3400 cm^−1^), C=O (1725 cm^−1^), C–O (1630 cm^−1^), C–OH (1225 cm^−1^), and C–O (1040 cm^−1^) functional groups (Figure S1a), indicating that various oxygen-containing groups were introduced. The XRD patterns of the prepared GO (Figure S1) show a strong and sharp diffraction peak at 2θ = 9.2°, which corresponds to a layer spacing of 0.97 nm and matches that of typical GO. Pure graphite has been reported to generate a diffraction peak at 2θ = 26° [42], but it was not observed in our measurement. The absence of this peak suggests that water molecules and OFGs (such as carboxyl, hydroxyl, and epoxy groups) were inserted in layers of graphite during the oxidation process, thus destroying the sp2 π-conjugated structure. Raman spectroscopy is an effective tool for the characterization of carbon materials. The Raman spectrum of our prepared GO shows two characteristic graphitic bands, the G band at around 1580 cm^−1^ and the D band at 1350 cm^−1^ (Figure S1). The G band is associated with sp^2^ C=C bond stretching, while the D band is due to the vibrations of sp^3^ carbon atoms. Typically, graphite shows strong and sharp G bands and insignificant D bands as a feature of highly graphitized materials with fewer defects. The Raman spectrum of our prepared GO shows significant D and G bands and the intensity ratio of ID/IG is 0.87. This result confirms that the chemical oxidation treatment destroyed the integrated layers and introduced large amounts of defects on the surface of graphite. According to TEM imaging characterization (see the inset of Figure S1b), the prepared GO is lamellar on the microscopic scale. The results indicated that the GO takes a multi-layer architecture through aggregation rather than being single-layered. GO dispersed in water was analyzed by the UV-Vis absorption method. Figure S1d shows the absorption spectrum of GO, which has the strongest absorption band present at 231 nm, due to the π→π* transition of the aromatic C=C bonds in the graphitic domain. A noticeable shoulder band appears around 310 nm, which can be assigned to n→π* transitions of various oxygen-containing functional groups, especially the carbonyl group. The UV-Vis absorption features of our GO are consistent with those reported in the literature [43].

### 2.2. Removal of TC Promoted by GO under Light

**Figure 1:**
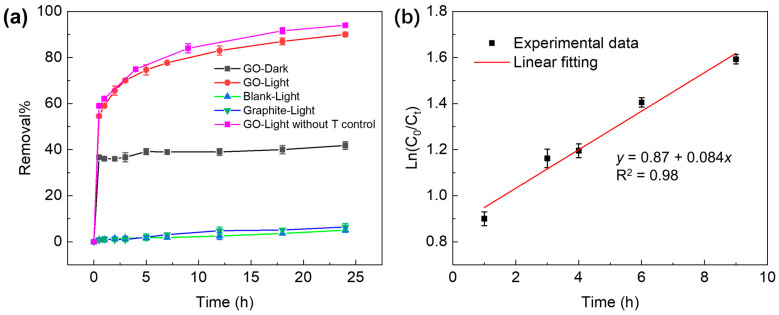
(**a**) TC removal by GO and graphite under dark or xenon lamp irradiation. (**b**) TC removal kinetic in the GO–light system. Initial conditions: [GO] = 200 mg·L^−1^, [TC] = 100 mg·L^−1^.

The results of GO-induced removal of TC under xenon lamp irradiation as well as relevant control experiments are shown in Figure 1a. The degradation rate of TC under light alone is insignificant. It can also be seen that the addition of natural graphite to TC solution does not cause much photodegradation of TC, indicating that graphite does not have any photocatalytic effect. Graphite contains only sp^2^ carbons in its structure and does not have effective adsorption sites for TC. Moreover, graphite has a zero band gap and, hence, cannot effectively induce any photocatalytic effects.

The TC removal curve of GO shows that 40% of TC in the test solution was removed within one hour under dark conditions. After one hour, the curve does not significantly change, indicating that the process has reached equilibrium. The rapid removal effect is because GO has a high specific surface area and a large number of active adsorption sites such as those oxygen-containing groups characterized by IR analysis. Under dark conditions, the removal of TC from water is primarily due to the adsorption of TC on the surface of GO. Our analysis shows that the equilibrium adsorption of TC by GO is about 235 mg·g^−1^, which is consistent with previous reports [40].

TC removal performance under light shows a stark contrast to that measured under dark conditions. As shown in Figure 1a (red color), the TC removal rate rapidly reaches 55% within one hour and then continues to increase. Eventually, the removal rate achieves 90%, which is more than double the value determined under dark conditions. The kinetics of TC removal under light irradiation without temperature control (pink color) showed a higher TC removal rate compared to that with the temperature maintained at room temperature (red color). A comparison of these results indicates that thermal effects delivered by the magnitude of the irradiance of the xenon light incident contribute to enhanced TC removal. In our experiments, the TC removal rate was determined by monitoring the absorbance at 356 nm, a characteristic absorption peak of TC. The removal kinetics of TC in this GO–light system is shown by the plot in Figure 1b. The linear correlation between Ln(*C*_0_/*C*_t_) and time in this plot discloses a first-order behavior.

To test the applicability of the GO–light system, the influence of various TC concentrations (30 to 200 mg·L^−1^) on the removal effect of TC was investigated. The removal rate of low-concentration TC (30 to 70 mg·L^−1^) in the GO–light system largely reached the maximum within one hour (Figure S2). The kinetics of TC removal in the GO–light system are well described by the Langmuir–Hinshelwood model expressed as Equation (1) [44]:

(1) $$\frac{1}{r_{0}}=\frac{1}{k_{r}}+\frac{1}{k_{r}k_{a}C_{0}}$$

where *r*_0_ is the initial reaction rate and *k_r_* is the reaction rate constant. *k_a_* is the equilibrium constant and *C*_0_ is the initial TC concentration. For the batch reactor, the initial reaction rate *r*_0_ can be obtained from the change of concentration in Equation (2) with time (the first 60 min in this work):

(2) $$\frac{dC}{dt}=-r$$

where *r* is the instantaneous removal rate of TC and *C* is the instantaneous concentration of TC.

**Figure 2:**
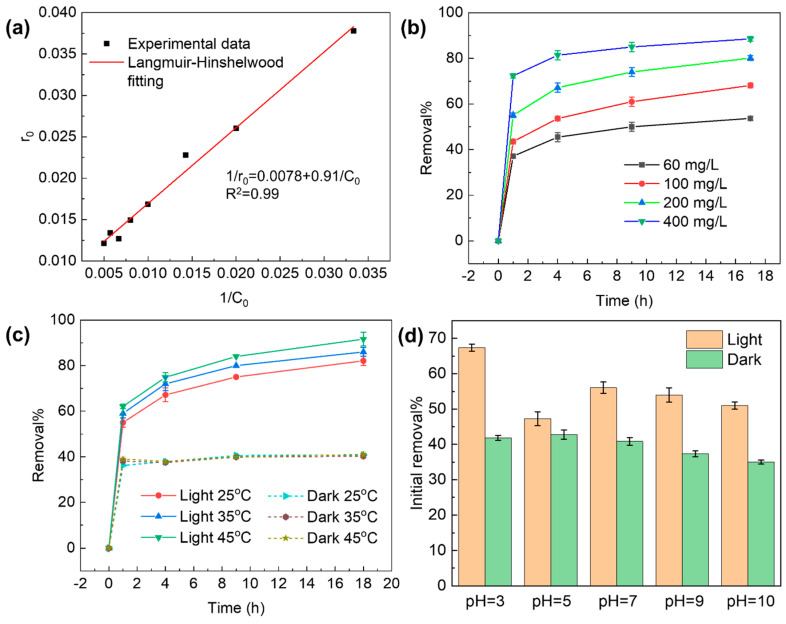
(**a**) Correlation of the TC initial concentration (35 to 200 mg·L^−1^) and *r*_0_ of the initial reaction rate by the Langmuir–Hinshelwood model. (**b**) Effect of GO dose on TC removal performance. (**c**) Effect of temperature on TC removal performance. (**d**) Removal of TC under dark and light conditions in different acidic and basic conditions (pH = 3–10).

Figure 2a shows the linear fitting of the kinetics data. The high correlation coefficient (R^2^ = 0.99) indicates that the photocatalytic removal of TC in the GO–light system can be well described by the Langmuir–Hinshelwood model. In addition, we evaluated the effect of GO dose (30, 60,100, 200, 400 mg·L^−1^) on TC removal efficiency at an initial TC concentration of 100 mg·L^−1^. As shown in Figure 2b, with a gradual increase in added GO, the efficiency of TC removal of the GO–light system increases proportionally, and the TC removal rate within one hour achieves 36%, 43%, 55%, and 73%, respectively. After one hour, the removal rate increases much less significantly as a function of time, indicating that the TC removal in this time domain is slow and approaches equilibrium. The increased removal rate can be attributed to a larger surface area and more active sites [45].

The effect of temperature on the performance of GO in TC removal is shown in Figure 2c. The effect of temperature on the adsorption removal of TC is small in dark conditions, but much larger in light. The removal rate of TC is also found to gradually increase with increasing temperature. When the temperature is changed from 25 to 45 °C, the removal rate of TC is increased by about 10% within one hour. It is likely that at elevated temperature, the surface of GO becomes more adsorptive for TC and the photocatalytic effects of GO are further enhanced.

The effect of pH on the removal of TC by the GO–light system is depicted in Figure 2d. Under dark conditions, the adsorption performance of GO toward TC gradually decreases with increasing pH. Under acidic conditions (pH < 3.3), TC is mainly positively charged in water, which can be captured by GO through the electrostatic attraction and hydrogen-bonding interactions. When the pH is above 3.3, TC changes into a zwitterionic or negatively charged form. The electrostatic attraction between TC and GO is accordingly reduced and can even be changed into electrostatic repulsion [40]. However, the removal rate of TC decreased by only 6.8% when the pH changed from 2 to 10. It is possible that other non-covalent forces, such as van der Waals, dipole interactions, and hydrogen bonding, come into play to compensate for the loss of electrostatic attraction at higher pH values. Under light, the effect of pH becomes more significant. At pH 3, the removal rate of TC reaches a maximum of 67%, which is 16% higher than that at pH 10. The results indicated that acidic conditions are more favorable for the removal of TC in the GO–light system.

### 2.3. Effects of Radical and Hole Inhibitors on TC Removal Efficiency and ESR Analysis

**Figure 3:**
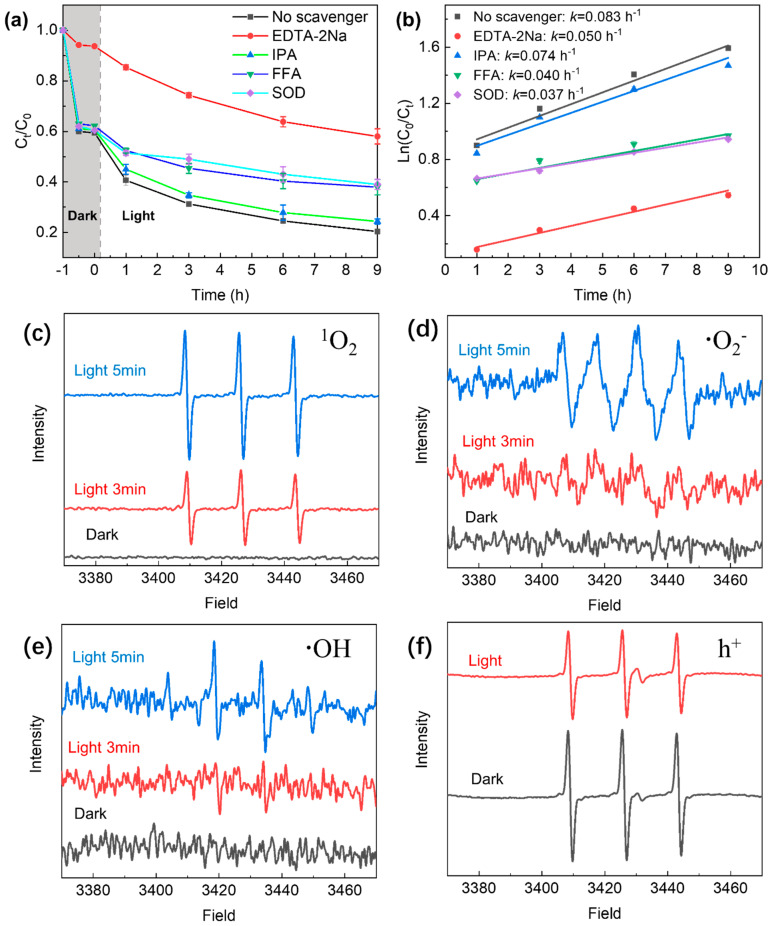
(**a**) Effect of various scavengers on TC removal in the GO–light system. (**b**) First-order kinetic plots for the scavenger-involved reactions. ESR spectra of free radicals and holes trapped by TEMP (^1^O_2_), TEMPO (h^+^) and DMPO (•O_2_^−^ and •OH) under xenon lamp irradiation: in aqueous dispersion for trapping ^1^O_2_ (**c**); in methanol dispersion for trapping •O_2_^−^ (**d**); in aqueous dispersion for trapping •OH (**e**); in aqueous dispersion for trapping h^+^ (**f**).

Studies have shown that GO is prone to photoreaction under light and may be involved in the production of electron–hole pairs [33]. In this study, radical scavenging experiments were performed to examine the mechanism of TC removal by the GO–light system. EDTA-2Na, FFA, SOD, and isopropyl alcohol were chosen as radical scavengers in our experiments, as they can effectively interact with the reactive species generated during photoactivation of GO; specifically, EDTA-2Na is a hole scavenger [46], FFA is a superoxide radical scavenger [47], SOD is a superoxide radical scavenger [48], and isopropyl alcohol is a hydroxyl radical scavenger [49]. As shown in Figure 3, the presence of EDTA-2Na results in a significant reduction in TC removal efficiency, confirming that holes are involved in the photocatalytic degradation of TC on GO. It is worth noting that EDTA-2Na was also found to show a somewhat inhibitive effect on TC removal under dark conditions (Figure 3a); however, the extent is not as significant as in light. The inhibitive effect observed in the dark can be explained by the fact that EDTA-2Na and TC make competitive adsorption on the GO surface, hence, attenuating TC removal efficiency. The significant inhibition of TC removal by EDTA-2Na under light can, therefore, be attributed to both the photogenerated holes in GO and the competitive adsorption of EDTA-2Na.

The presence of FFA and SOD also significantly inhibits the removal of TC, attesting to the fact that singlet oxygen (^1^O_2_) and the superoxide radical (•O_2_^−^) play important roles in the degradation of TC under irradiation. However, the introduction of isopropyl alcohol (hydroxyl radical scavenger) to the system does not result in significantly inhibited TC removal. It is, therefore, reasonable to conclude that hydroxyl radicals are not significantly produced during the photoexcitation of GO.

The reactive oxygen species (ROS) involved in the photodegradation of TC were investigated by ESR analysis. In this work, DMPO was used as a spin trap to detect hydroxyl radicals and superoxide radicals, TEMP was used to detect singlet oxygen, and TEMPO was used to detect holes [50,51]. As shown in Figure 3c, the TEMP–^1^O_2_ signal was recorded by ESR after GO was irradiated by a xenon lamp in water. The signals acquired after 5 min irradiation are of much stronger intensity than those after 3 min irradiation. The presence of •O_2_^−^ was also detected. In Figure 3d, the signals of •O_2_^−^ show significant enhancement in intensity when the irradiation time is increased from 3 min to 5 min. These results suggest that ^1^O_2_ and •O_2_^−^ are the active species involved in the removal of TC by the GO–light system. In our ESR analysis, •OH signals were also detected, but they were not particularly obvious and only observable from the noisy baseline (Figure 3e). The weak signals suggest that •OH is not sufficiently produced through photoexcitation of the GO, and, hence, plays only a minor role in the TC removal process. TEMPO can produce a triplet ESR signal by itself, and this ESR signal intensity would be weakened by reaction with h^+^, forming spin-adducts of (TEMPO–h^+^) [52]. In other words, the weakening of TEMPO signals indicates the generation of h^+^. As shown in Figure 3f, the intensity of the peak produced by TEMPO noticeably decreased after light irradiation, corroborating that holes are generated in the GO–light system. Overall, the results of our ESR studies agree with the different inhibitory removal effects observed for EDTA-2Na, FFA, SOD, and isopropyl alcohol on TC as shown in Figure 3a.

**Figure 4:**
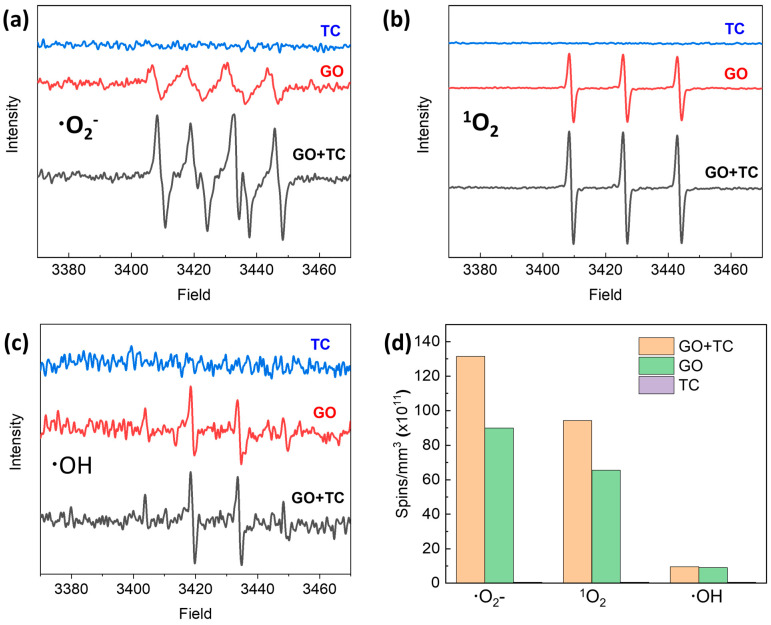
ESR spectra of radicals trapped by DMPO (•O_2_^−^ and •OH), TEMP (^1^O_2_), and TEMPO (h^+^) in GO aqueous dispersion under xenon lamp irradiation: (**a**) for trapping ^1^O_2_, (**b**) for trapping •O_2_^−^, (**c**) for trapping h^+^, and (**d**) quantitative comparison of •O_2_^−^, •OH, and ^1^O_2_ produced by GO, TC, and [GO + TC], respectively.

Studies have demonstrated that the TC molecule is sensitive to various light sources in aqueous solutions [53,54,55]. For example, Hu et al. reported that TC can be effectively photodegraded on the surface of TiO_2_ under visible light irradiation. This reactivity was rationalized by the fact that TC with photosensitivity may form a complex with the surface of TiO_2_ to generate superoxide free radicals through the absorption of visible light [55]. In our work, the ESR spin-trap technique was used to analyze the production of ROS in TC solution by GO under light irradiation. As shown in Figure 4, both ^1^O_2_ and •O_2_^−^ were noticeably detected under light conditions. Interestingly, the concentration of ^1^O_2_ and •O_2_^−^ generated by GO in TC solution increased by about 1/3 compared with that in clean water, while the generation of •OH remained largely unchanged compared with that in clean water (Figure 4d). No ROS were detected in the TC solution in the absence of GO under the same light conditions. These results suggest that the interactions of GO and TC under photoirradiation contribute to increased ^1^O_2_ and •O_2_^−^ generation. GO has a large surface area and contains abundant oxygen-containing groups and sp^2^ carbon domains on its surface to give strong adsorption performance for TC [40]. The adsorption of TC molecules on GO is facilitated by various non-covalent forces (e.g., electrostatic attraction, van der Waals forces, and hydrogen-bonding interactions). TC is a photosensitive molecule. Upon photoirradiation, the excited-state TC can inject electrons to the conduction band of GO, which in turn promotes the formation of more ^1^O_2_ and •O_2_^−^.

### 2.4. TC Degradation Mechanism in GO–Light System

**Figure 5:**
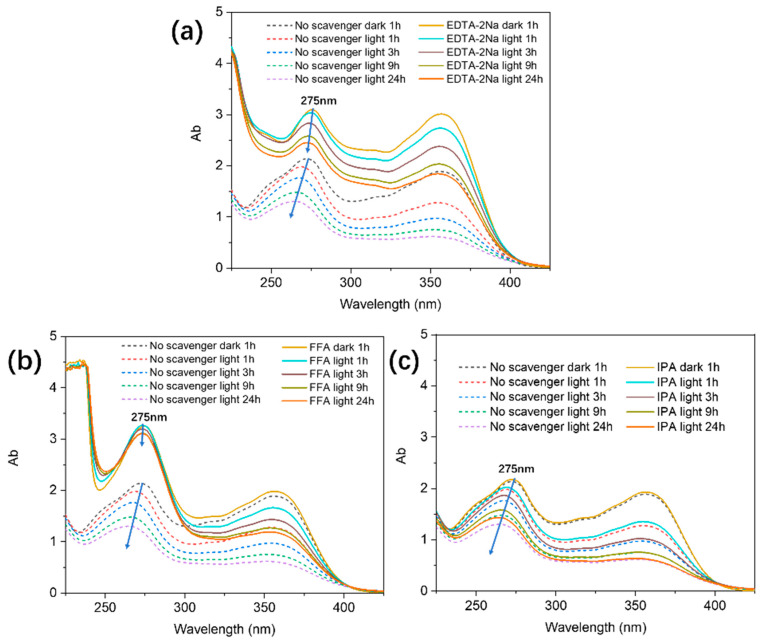
UV-Vis absorption spectra of TC solutions at different time intervals: (**a**) the effect of EDTA-2Na, (**b**) the effect of FFA, and (**c**) the effect of IPA.

Aqueous samples of TC were analyzed by UV-Vis in TC solutions treated by GO (see Figure 5). The absorption spectra show that TC generates two main absorption peaks at 275 nm and 357 nm. The absorption at 357 nm is due to the aromatic rings B-D in TC, including enols and developed chromophores. The absorption at 275 nm is mainly related to the acyl-amino and hydroxyl groups on aromatic ring A [56]. These characteristic absorption bands were found to gradually decrease with increasing reaction time, suggesting that TC was gradually removed from aqueous solution. Compared to dark conditions, the absorption peak at 275 nm under photoirradiation shows a significant blue shift (Figure 5) as the reaction progresses. This observation is indicative of degradation of the TC structure, and the degradation of TC most likely occurred on the aromatic ring A part due to the attack by the generated ROS (e.g., ^1^O_2_, •O_2_^−^) and the reaction with holes. In fact, in the radical scavenging experiments, the addition of ^1^O_2_ and hole scavengers significantly inhibited the blue shift of the 275 nm absorption peak (Figure 5b,c), whereas the addition of hydroxyl radical traps little inhibited the blue shift of the peak (Figure 5d). These experimental results further confirm that ^1^O_2_ and holes directly participate in the photocatalytic degradation of TC, but hydroxyl radical is only minorly involved.

**Figure 6:**
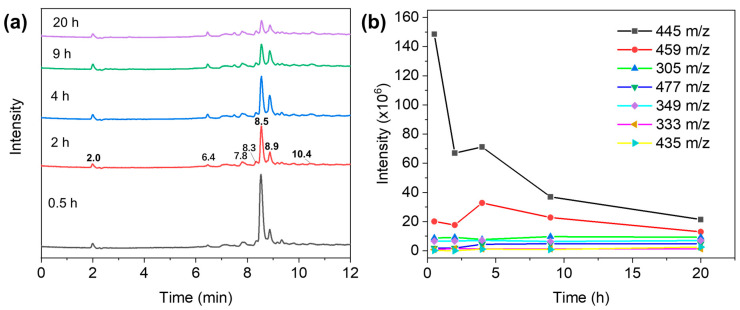
(**a**) Total ion current LC-MS/MS chromatogram of TC solution treated by GO irradiation at different time points. (**b**) Product evolution of TC during the removal of TC process by the GO–light system.

To shed more light on the mechanism of TC degradation by the GO–light system, the intermediate products of TC during GO–light treatment were evaluated by liquid chromatography–electrospray ionization–tandem mass spectrometry (LC-ESIMS/MS) analysis. Figure 6a shows the total ion current LC-MS/MS chromatogram of TC treated by the GO–light system at different times. Based on the MS analysis, seven different kinds of products can be identified at *m*/*z* = 477 (retention time 6.4 min), 305 (retention time 7.8 min), 349 (retention time 8.3 min), 459 (retention time 8.9 min), 333 (retention time 10.4 min), and 435 (retention time 10.4 min), respectively. The detailed mass spectra corresponding to each chromatographic peak are given in Figure S4. The possible degradation products of TC are proposed in Table S2, where *m*/*z* = 445 (retention time 8.5 min) is taken as the parent TC.

As shown in Figure 6a, the amount of TC gradually decreases with increasing GO–light treatment time. Among the TC photocatalytic degradation products, the abundance of *m*/*z* = 459 product is the most obvious, indicating that this intermediate is the main degradation product in the process of TC removal by the GO–light system. Product *m*/*z* = 459 can be obtained by introducing two carbonyl groups to the *para*-positions of the TC aromatic ring in the presence of singlet oxygen and superoxide radicals [57,58]. With increasing treatment time, the concentration of the intermediate shows a trend of first increasing and then decreasing (Figure 6b). This can be explained by that a large amount of the TC present in the early stage of GO–light treatment is degraded into *m*/*z* 459 by ROS produced in the system, and the intermediate may be further degraded through adsorption or remain on the GO surface as the reaction proceeds. The double bond at the C11a-C12 position of TC may cause C11a to be hydroxylated by •OH because the electron-withdrawing group is more vulnerable to radical attack. In addition, hydroxylation may occur at the CH_3_-NR site, leading to the production of the *m*/*z* = 477 product [58,59].

**Figure 7:**
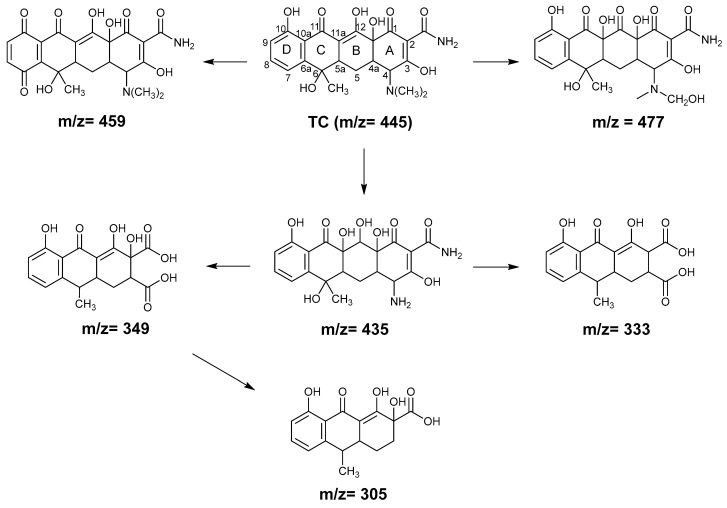
Proposed photodegradation pathways and the degraded products of TC by the GO–light system.

The *m*/*z* = 435 degradation products can be formed by demethylation of dimethyl amine of TC under ^1^O_2_ and •O_2_^−^ attack and hydroxylation destruction under H_2_O_2_/H_2_O attack [28,60,61,62]. The carbon–carbon double bond on *m*/*z* = 435 can be attacked by ROS, and ring A is subsequently cracked during photocatalytic oxidation to produce intermediate *m*/*z* = 349 [63]. A similar reaction mechanism can be introduced to form the intermediate *m*/*z* = 333 [64]. The product *m*/*z* = 349 can be further decarboxylated to form another product *m*/*z* = 305 [63]. Notably, there is also a distinct peak at the retention time of 2.0 min in the LC-MS chromatogram, which could be attributed to the partial dissolution of GO in water, as the same chromatogram peak and the same mass spectra (Figure S5) appear at the retention time of 2.0 min under dark conditions. Combining our experimental outcomes and literature results, we arrive at the possible reaction pathways and the degraded products of TC degradation by the GO–light system proposed in Figure 7.

**Figure 8:**
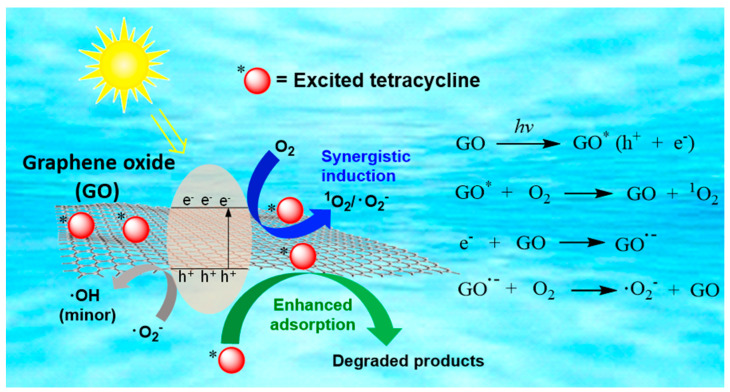
The proposed mechanism of TC degradation by a GO–light system (red circle with star symbol represents excited tetracycline).

The DFT method was used to reveal the adsorption property of TC on GO under light irradiation (Figure S6). Because TC is a photosensitive molecule, excited-state TC was used as a model for the GO–light system. The binding energies of GO with the excited-state TC and ground-state TC were calculated to be −4.91 ev and −2.31 ev, respectively. The theoretical results indicated that TC can adsorb more easily on the GO surface under light irradiation compared to under dark conditions, which also contributed to the enhanced removal rate of TC by the GO–light system. The possible removal mechanism of TC by GO under irradiation is illustrated in Figure 8. As a semiconductor-like material, GO could generate oxidative (valence band holes, h^+^) and reductive (conduction band electron, e^−^) transient species when exposed to sunlight. In addition, sunlight irradiation should change GO from the ground state to the excited state (GO*), which in turn induces the formation of singlet oxygen (^1^O_2_) through the energy transfer between GO* and dissolved oxygen in water [65]. The photoinduced electrons could quickly migrate to the surface of GO. The abundant oxygen functional groups (OFGs) on the GO surface facilitate the generation of GO radical anions and subsequent formation of ·O_2_^−^ [66]. The migration of electrons can improve the separation efficiency of photogenerated e^−^–h^+^ pairs and enhance the h^+^-dominated direct oxidation on TC reaction [67]. It is also possible that hydroxy radicals (·OH) might be produced from ·O_2_^−^ [68], but ·OH plays only a minor role in photo-enhanced TC removal in the presence of GO.

From a fundamental perspective, the structural homogeneity of GO is an important factor in its photocatalytic effects on generating ROS and degrading organic substances on its surface. Gaining a deeper insight into this aspect, however, requires a more carefully controlled synthesis of GO and thorough characterizations of the structure and composition of GO. It is also worth noting that the molecular structures of tetracycline and many other antibiotics contain stereogenic centers and, therefore, show optical activity. The use of polarized light to induce selective photoexcitation and subsequent degradation on the surface of GO represents another intriguing direction for future work. Moreover, tuning the nonlinear optical effects of GO may create a new avenue for optimizing and enhancing the performance of GO in combination with other functional nanomaterials (e.g., metal and metal oxide nanoparticles) [69]. Studies along these directions are warranted for future work to further improve the performance of our GO-based photocatalytic systems.

## 3. Materials and Methods

### 3.1. Chemical Reagents

Detailed information on the chemical reagents used in this study is provided in Text S1.

### 3.2. Preparation of GO

GO was prepared from natural graphite powder using an improved Hummers method (see Text S2 for more details) [70]. The characterization methods of GO are presented in Text S3.

### 3.3. Removal of TC under Light Condition

A xenon lamp (300 W) was used as a simulated sunlight source, and the distance between TC solution and light source was kept at 10 cm. The conical flask containing TC solution and GO sample was placed in a water bath and was blown with a fan to maintain the sample solution at room temperature (25 °C) during the TC removal test. An experiment that was conducted under the same conditions without the presence of GO was used as a control. Further, an experiment in which a conical flask containing tetracycline solution and GO sample was directly irradiated by xenon light without temperature control was also conducted and the results were used to evaluate the thermal effect of the light irradiation. A typical TC removal experiment was carried out through the following steps: 10 mg of GO was added to a 50 mL solution of TC (100 mg·L^−1^). The resulting suspension was stirred under irradiation or dark conditions for a certain period of time. After that, the sample was centrifuged and aliquots from the top of the solution were collected for analysis. In order to study the effect of pH on TC removal, the pH values of TC solutions were adjusted in the range of 3.0–10.0, using a small amount of 0.1 M hydrochloric acid or 0.1 M sodium hydroxide solution. Ethylenediaminetetraacetic acid disodium salt (EDTA-2Na), furfuryl alcohol (FFA), Superoxide dismutase (SOD), and isopropanol (IPA), were used as holes, ^1^O_2_, •O_2_^−^, and •OH scavengers, respectively. All experiments were performed in triplicate.

### 3.4. Analytical Methods

The concentration of TC in solution of each sample was determined by the UV-Vis absorption method. The TC removal rate (*R*) at a time (t) can be calculated using the following formula:

(3) $$R=\frac{C_{0}-C_{t}}{C_{0}}$$

where *C*_0_ and *C_t_* are the initial concentration of TC and the concentration of TC at time t, respectively.

Electron spin resonance (ESR) experiments were performed using a spectrometer (Brucker EMX, Germany) equipped with a 300 W xenon lamp. The test conditions for •OH, ^1^O_2_, •O_2_^−^, and holes are given in Text S4. Photodegradation intermediates of TC were detected using an HPLC–MS system (Agilent 1290/6460, Triple Quad MS). Detailed information on the HPLC–MS system analysis is given in Text S5. Detailed information on the computational modeling is provided in Text S6.

## 4. Conclusions

The removal of TC from water by the GO–light system has been systematically studied. Our results show that light irradiation significantly enhances the removal of TC. More than 90% of TC was removed within 20 h (100 mg·L^−1^ TC, 200 mg·L^−1^ GO) under light irradiation, which is 50% more efficient than the action of GO in dark conditions. Radical scavenging experiments, ESR, and UV-Vis spectral analysis all point to the fact that ^1^O_2_, •O_2_^−^, and h^+^ are responsible for the enhanced TC removal. The degradation pathway of TC is proposed based on the intermediates identified by the LC-ESIMS/MS technique. The studies provide experimental and theoretical support for the development of a new strategy for the removal of pollutants from water. At the same time, recycling or isolating GO after water treatment is still a challenge as GO is highly dispersible in water due to its abundant oxygen functional groups. This challenge is expected to be overcome by designing GO composites that can be readily isolated out of water and retain the performance of GO in terms of excellent absorptivity and photoactivity. Overall, we envision the concept of enhanced adsorption and synergistic induction of ROS generation will find extensive applications in sustainable materials and environmental technologies.

## Data Availability

Data are contained within the article and Supplementary Materials.

## Supplementary Materials

The following supporting information can be downloaded at: https://www.mdpi.com/article/10.3390/molecules29163765/s1. Text S1. Chemical reagents; Text S2. Preparation and characterization of GO; Text S3–S6. Detailed information of analytic methods; TC removal model; ESR spectra; UV-Vis spectra; Total ion current LC-MS/MS chromatogram analysis during TC removal and corresponding mass spectra; Proposed intermediate structures; Computational modeling. Figure S1. (a) FTIR spectrum of prepared GO; (b) XRD spectrum and TEM image of GO. (c) UV-VIS absorption spectrum of GO suspended in water. Figure S2. The influence of various TC concentrations (30 to 200 mg·L^−1^) on the removal effect of TC by the GO-Light system. Table S1. First order reaction kinetics of tetracycline removal by GO-Light system in the presence of various radical scavengers. Figure S3. UV–vis absorption spectra of TC solutions at different time intervals under light and dark conditions. Figure S4. Mass spectra corresponding to each chromatography peak in the total ion current LC-MS/MS chromatogram of tetracycline solution treated by GO under light. (2 h as a typical example). Table S2. The identified of TC and its possible transformation products during the photocatalysis. Figure S5. (a) Total ion current LC-MS/MS chromatogram of tetracycline solution treated by GO under dark and light conditions; GO-Light control is the pure water treated by GO under light. (b) Mass spectra collected at retention time of 2.1 min. Figure S6. Configuration for TC and GO structure interaction, references [71,72,73,74,75,76].
