# Supplementary material for: Mapping Photogenerated Electron–Hole Behavior of Graphene Oxide: Insight into a New Mechanism of Photosensitive Pollutant Degradation
Source: Molecules. 2024 Aug 8;29(16):3765. doi: 10.3390/molecules29163765 (PMC11357050; doi:10.3390/molecules29163765)
Supplement: Supplementary file 1 [file molecules-29-03765-s001.zip › molecules-3123228-supplementary.pdf]

molecules

Mapping Photogenerated Electron–Hole Behavior of Graphene Oxide: Insight into a New Mechanism of Photosensitive Pollutant Degradation

Kaijie Ni [1,*], Yanlong Chen [1], Ruiqi Xu [1], Yuming Zhao [2] and Ming Guo [1,*]

[1] College of Chemistry and Materials Engineering, Zhejiang Agriculture and Forestry University, Hangzhou 311300, China
[2] Department of Chemistry, Memorial University of Newfoundland, St. John's, NL A1B 3X7, Canada
* Correspondence: nikaijie@zafu.edu.cn (K.N.); guoming@zafu.edu.cn (M.G.)

Text S1. Chemical reagents

Graphite, TC hydrochloride, ethylenediaminetetraacetic acid disodium salt (EDTA-2Na), isopropanol (IPA), furfuryl alcohol (FFA), Superoxide dismutase (SOD), 4-Hydroxy-2,2,6,6-tetramethyl-peperidine (TEMP), 2,2,6,6-tetramethylpiperidine-1-oxyl (TEMPO) and 5,5-Dimethyl-1-pyrroline-N-oxide (DMPO) were purchased from Macklin (Shanghai, China). $NaNO_3$, H_2SO_4 (98 wt%), $KMnO_4$, H_2O_2 (30 wt%), HCl (37 wt%) were purchased from Shanghai Linfeng Chemical Reagent Co., Ltd. All solvents used in the experiments are of analytical grade and used without further purification. Ultrapure water with a resistivity of 10-16 MΩ cm from Water Purification System (Shanghai Leading Water Treatment Equipment, China) was used throughout this work.

Text S2. Preparation of graphene oxide (GO)

An appropriate amount of concentrated sulfuric acid was added to a round-bottom flask placed in an ice bath. Graphite powder (2.00 g) and sodium nitrate (1.00 g) were added while stirring. Potassium permanganate (6.00 g) was added in small portions over 20 min and stirred for 20 min below 20 ^{o}C. Then the temperature was increased to 35 ^{o}C, and the mixture was stirred for another 20 min. Deionized water (100 mL) was added slowly and the mixture was further stirred for 30 min. After the oxidation reaction was complete, an appropriate amount of H_2O_2 aqueous solution (50 mL, 30%) was added to quench excess potassium permanganate. The reaction solution turned to a bright yellow color. The reaction mixture was settled and the supernatant was poured out. The collected solids were crude GO, which was washed with 5% hydrochloric acid solution and deionized water until no sulfate ions were detected in the filtrate. Finally, the resulting GO products were subjected to freeze-drying.

Text S3. Characterization methods

Fourier transfer infrared (FT-IR) spectral analysis was performed using a Shimadzu Prestege21 spectrophotometer. Scanning electron microscopic (SEM) imaging was performed on a Hitachi Regulus 8100 scanning electron microscope. Transmission electron microscopic (TEM) imaging was performed on an FEI Tecnai-F30 instrument. Freeze-drying using a Dongfeng-101 freeze-drying machine (Shanghai Lingke Industrial Development Co., Ltd.). UV-visible absorption spectral analysis (UV-VIS) was conducted on a UV-3600 spectrophotometer (Shimadzu, Japan). Electron spin resonance (ESR) experiments were performed in a spectrometer (Brucker EMX, Germany) equipped with a 300W xenon lamp. Photodegradation intermediates of TC were detected by an HPLC–MS system (Agilent 1290/6460, Triple Quad MS).

Text S4. Electron spin resonance method

In each experiment, a sample in borosilicate glass capillary was placed into a quartz tube and irradiated with the xenon lamp in the cavity of the ESR instrument. DMPO (200 mM) was added prior to the experiment to trap •OH and $•O_2^-$ where sample solution is added to water/methanol system. ESR signals was acquired at 3 min and 5 min, respectively. TEMP (200 mM) was added prior to the reaction to trap $^1O_2$ where sample solution was added to water system and ESR signals was determined at 3 min and 5 min, respectively. The sample concentrations of GO and TC were 200 mg L^{-1} and 100 mg L^{-1}, respectively.

Text S5. Liquid chromatography high resolution mass spectrometer method

HPLC–MS system was equipped with a Zorbax Eclipse Plus C18 column (3.0×150 mm, 1.8 micron). The mobile phase was a mixture of 15% methanol and 85% formic acid (1‰) with a flow rate of 0.3 mL min^{-1}. The column temperature was controlled at 25 °C. MS was performed by operating in the positive ion mode using ESI under the following conditions: capillary voltage 3000 V (positive), 3500 V (negative); nozzle voltage 500 V; Nebulizer 45 psi; Temperature of gas 350 °C; Gas flow 5 L min^{-1}; Temperature of sheath gas 250 °C, sheath gas flow 11 L min^{-1}. MS spectra were obtained by scanning the mass range from 50 to 1000 m/z.

Text S6. Computational modelling

The geometries of TC molecule and GO at ground state were optimized with dispersion corrected density functional theory (DFT-D3) at the PBE0-D3/def2-SVP[1, 2] level using Gaussian program. The excited molecular structures were also calculated at the PBE0-D3/def2-SVP level with the time-dependent density functional theory (TDDFT) method. All these DFT calculations were performed using Gaussian 16 program suite (Gaussian, Inc., Wallingford CT, 2016). The binding energy of the complex was calculated from the formular:

$$E(bind) = E(M + GO) - E(M) - E(GO) \qquad (S1)$$

where E(M) and E(GO) is the energy of isolated tetracycline molecule and graphene oxide, E(M+GO) is the total energy of the complex structure.

For the complex of TC adsorbed on GO, one hundred possible initial structures were generated using genmer program and then searched using molclus program[3]. These clusters were optimized using xTB program[4] with semi-empirical method GFN2-xTB[5, 6] firstly. After the preliminary structural optimization, the cluster with the lowest energy was selected as the optimal structure that is shown in Figure S6.

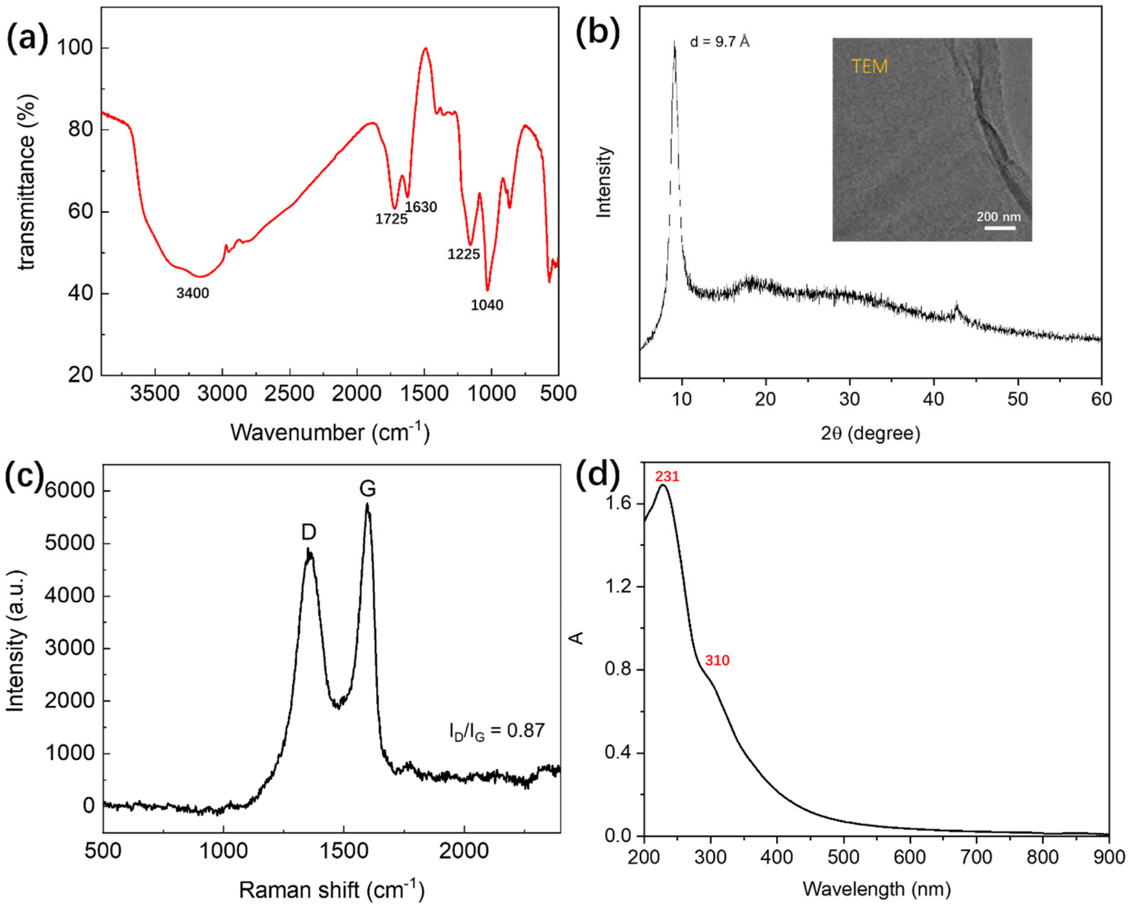

Figure S1. (a) FTIR spectrum of prepared GO; (b) XRD spectrum and TEM image of GO. (c) UV-VIS absorption spectrum of GO suspended in water.

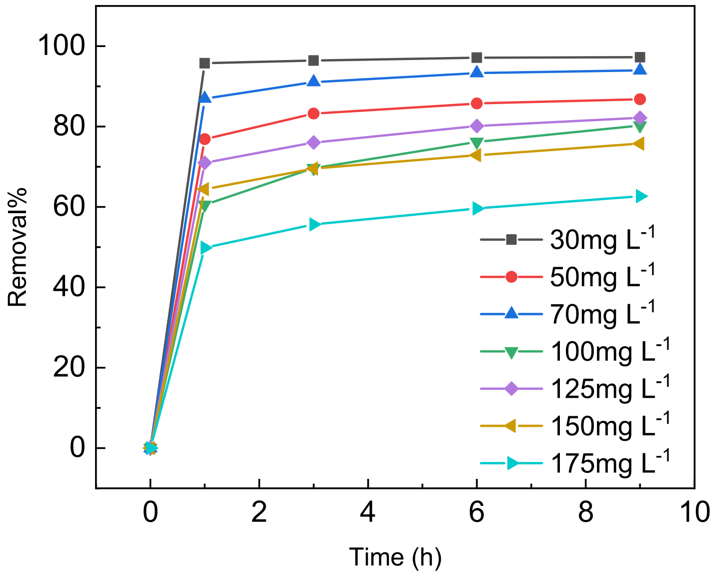

Figure S2. The influence of various TC concentrations (30 to 200 mg·L-1) on the removal effect of TC by the GO-Light system.

Table S1. First order reaction kinetics of tetracycline removal by GO-Light system in the presence of various radical scavengers.

Scavenger	Intercept	Slope	R^2
No scavenger	0.859	0.083	0.98
EDTA-2Na	0.125	0.050	0.97
IPA	0.818	0.078	0.96
FFA	0.619	0.040	0.97
N_2	0.626	0.037	0.99

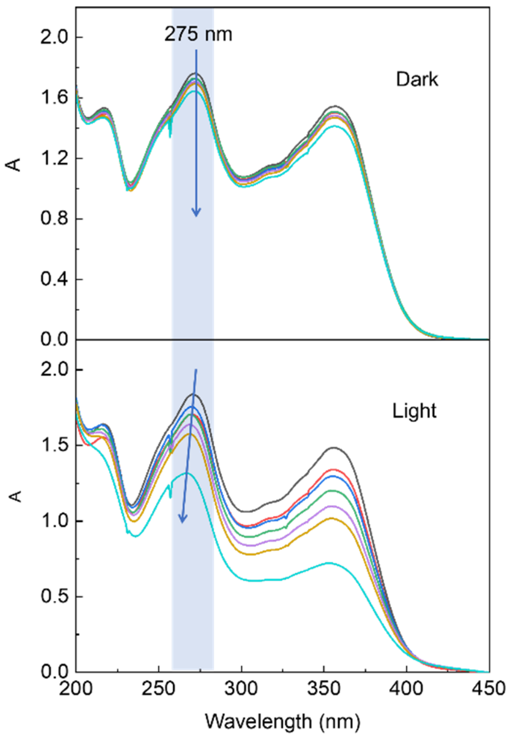

Figure S3. UV–vis absorption spectra of TC solutions at different time intervals under light and dark conditions.

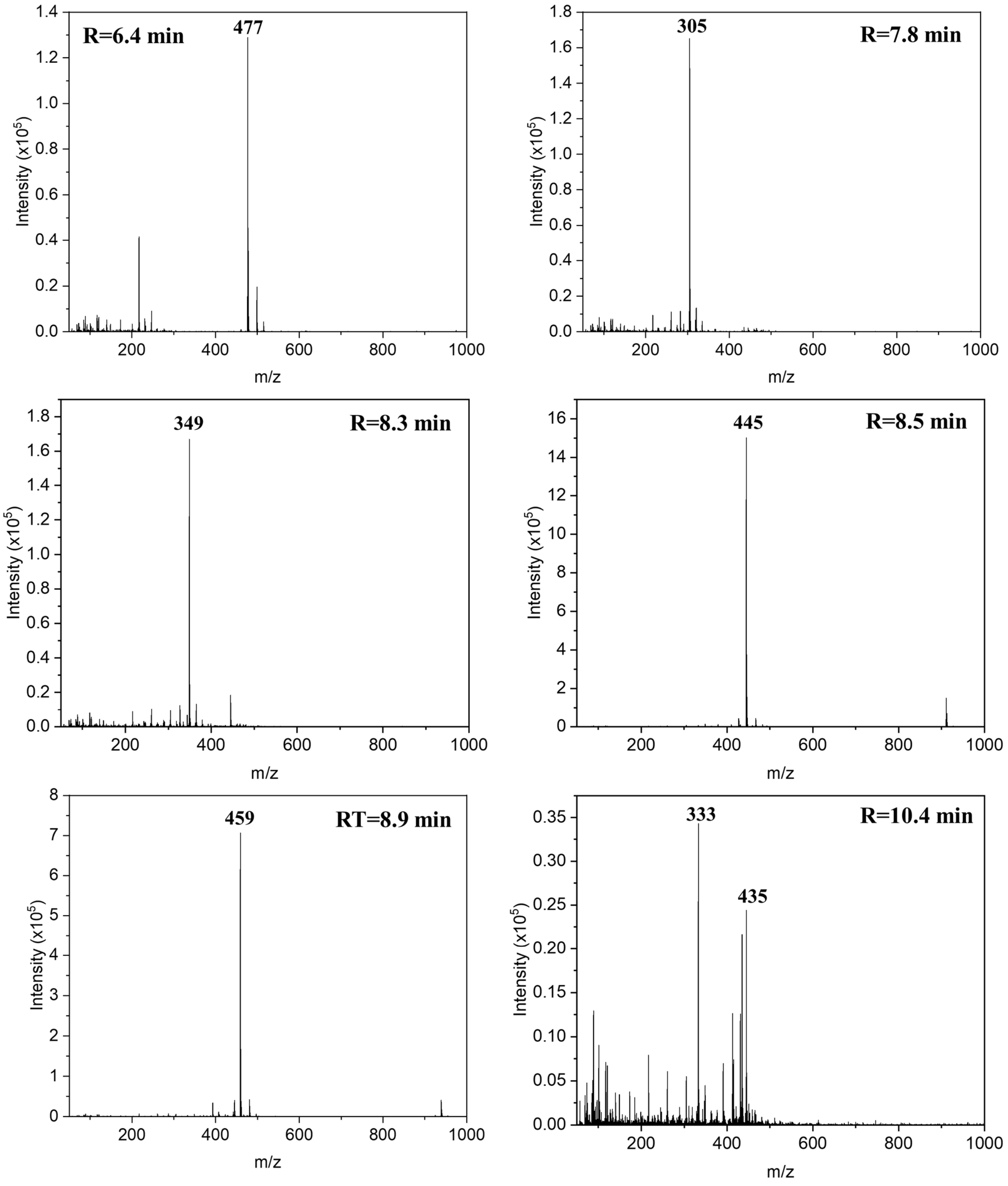

Figure S4. Mass spectra corresponding to each chromatography peak in the total ion current LC-MS/MS chromatogram of tetracycline solution treated by GO under light. (2 h as a typical example).

Table S2. The identified of TC and its possible transformation products during the photocatalysis.

Compounds	RT (min)	Measured mass (m/z)	Proposed structure
TC	8.5	445	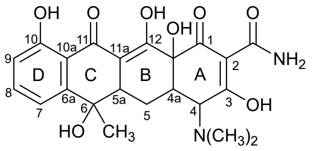
1	6.4	477	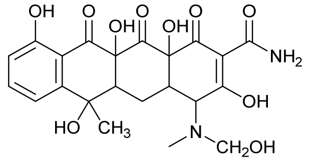
2	7.8	305	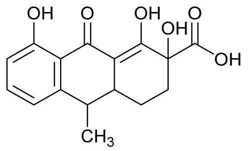
3	8.3	349	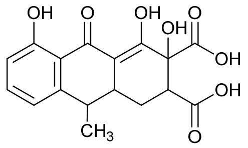
4	8.9	459	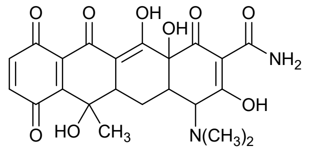
5	10.4	333	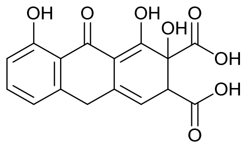
6	10.5	435	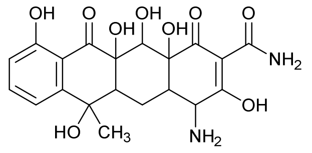

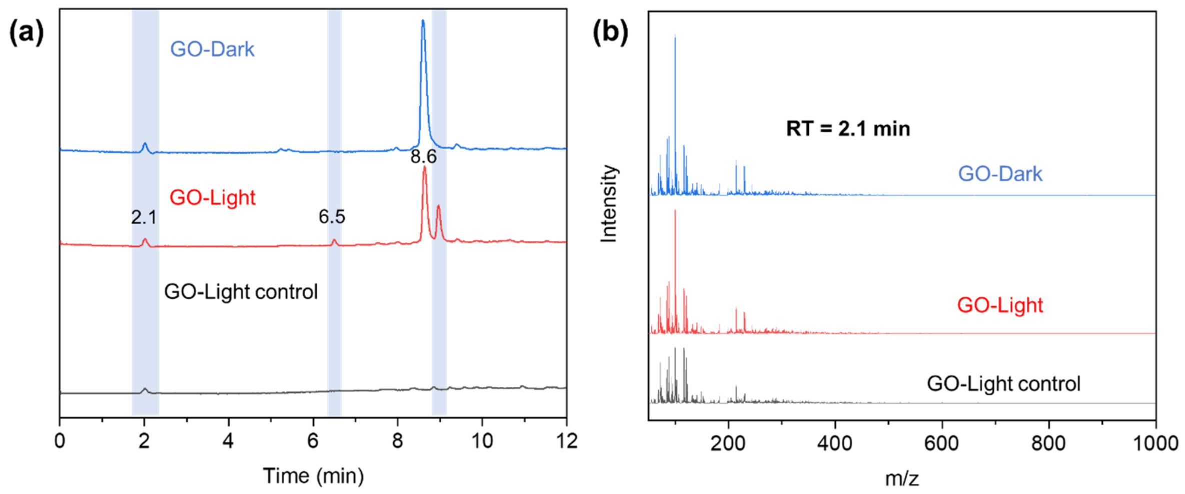

Figure S5. (a) Total ion current LC-MS/MS chromatogram of tetracycline solution treated by GO under dark and light conditions; GO-Light control is the pure water treated by GO under light. (b) Mass spectra collected at retention time of 2.1 min.

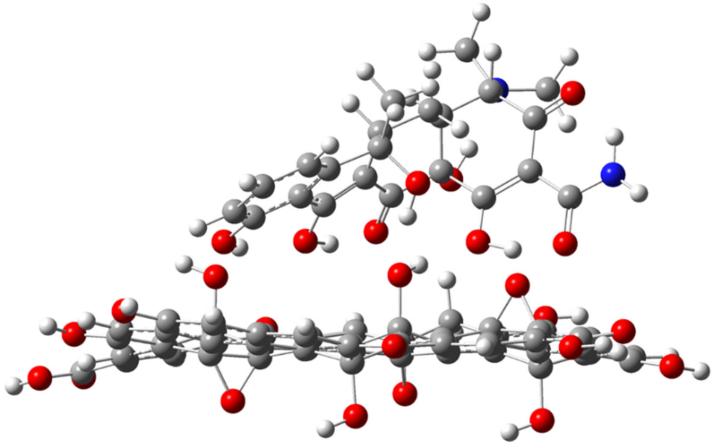

Figure S6. Configuration for TC and GO structure interaction.

References

1. Adamo, C.; Barone, V., Toward reliable density functional methods without adjustable parameters: The PBE0 model. *The Journal of Chemical Physics* **1999,** *110* (13), 6158-6170.

2. Weigend, F.; Ahlrichs, R., Balanced basis sets of split valence, triple zeta valence and quadruple zeta valence quality for H to Rn: Design and assessment of accuracy. *Physical Chemistry Chemical Physics* **2005,** *7* (18), 3297-3305.

3. Tian Lu, molclus program, Version 1.9.9.2, http://www.keinsci.com/research/molclus.html.

4. Bannwarth, C.; Caldeweyher, E.; Ehlert, S.; Hansen, A.; Pracht, P.; Seibert, J.; Spicher, S.; Grimme, S., Extended tight-binding quantum chemistry methods. *WIREs Computational Molecular Science* **2021,** *11* (2), e1493.

5. Grimme, S.; Bannwarth, C.; Shushkov, P., A Robust and Accurate tight-binding quantum chemical method for structures, vibrational frequencies, and noncovalent interactions of large molecular systems parametrized for all spd-block elements (Z = 1–86). *Journal of Chemical Theory and Computation* **2017,** *13* (5), 1989-2009.

6. Bannwarth, C.; Ehlert, S.; Grimme, S., GFN2-xTB—An accurate and broadly parametrized self-consistent tight-binding quantum chemical method with multipole electrostatics and density-dependent dispersion contributions. *Journal of Chemical Theory and Computation* **2019,** *15* (3), 1652-1671.
